# Supplementary material for: Pevonedistat Inhibits SOX2 Expression and Sphere Formation but Also Drives the Induction of Terminal Differentiation Markers and Apoptosis within Arsenite-Transformed Urothelial Cells
Source: Int J Mol Sci. 2023 May 23;24(11):9149. doi: 10.3390/ijms24119149 (PMC10252886; doi:10.3390/ijms24119149)
Supplement: Supplementary file 1 [file ijms-24-09149-s001.zip › ijms-2409901-supplementary.pdf]

Figure S1. Uncropped Western blots for Figures 2, 8, and 9

A. Uncropped blot for Fig. 2

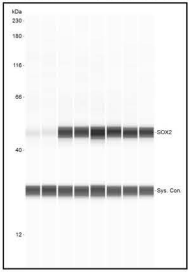

Lane 1&2: URO Par
Lane 3&4: URO As_I
Lane 5&6: URO As_II
Lane 7&8: URO As_III

B. Uncropped blot for Fig. 8

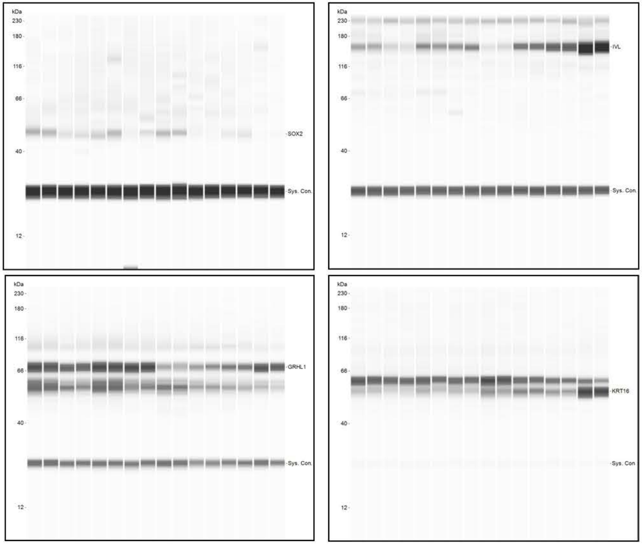

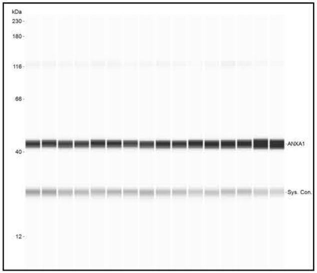

Lane 1&2: URO Par_DMSO
Lane 3&4: URO Par_PVD
Lane 5&6: URO Par_CIS
Lane 7&8: URO Par_PVD+CIS
Lane 9&10: URO As_I_DMSO
Lane 11&12: URO As_I_PVD
Lane 13&14: URO_As_I_CIS
Lane 15&16: URO_As_I_PVD+CIS

C. Uncropped blot for Fig. 9

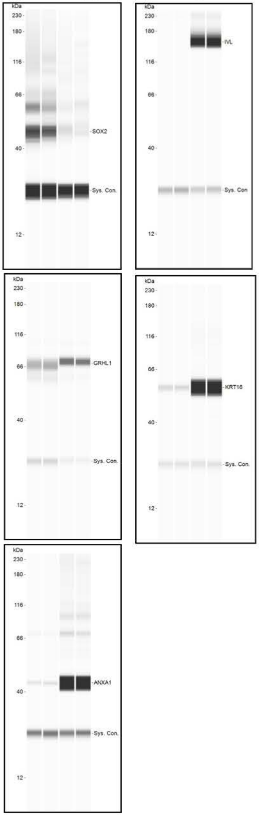

Lane 1&2: URO As_I
Lane 3&4: URO As_I_sphere

Figure S2. Proteomic analysis of UROtsa cells after 5 passages in 1 µM As^{3+}

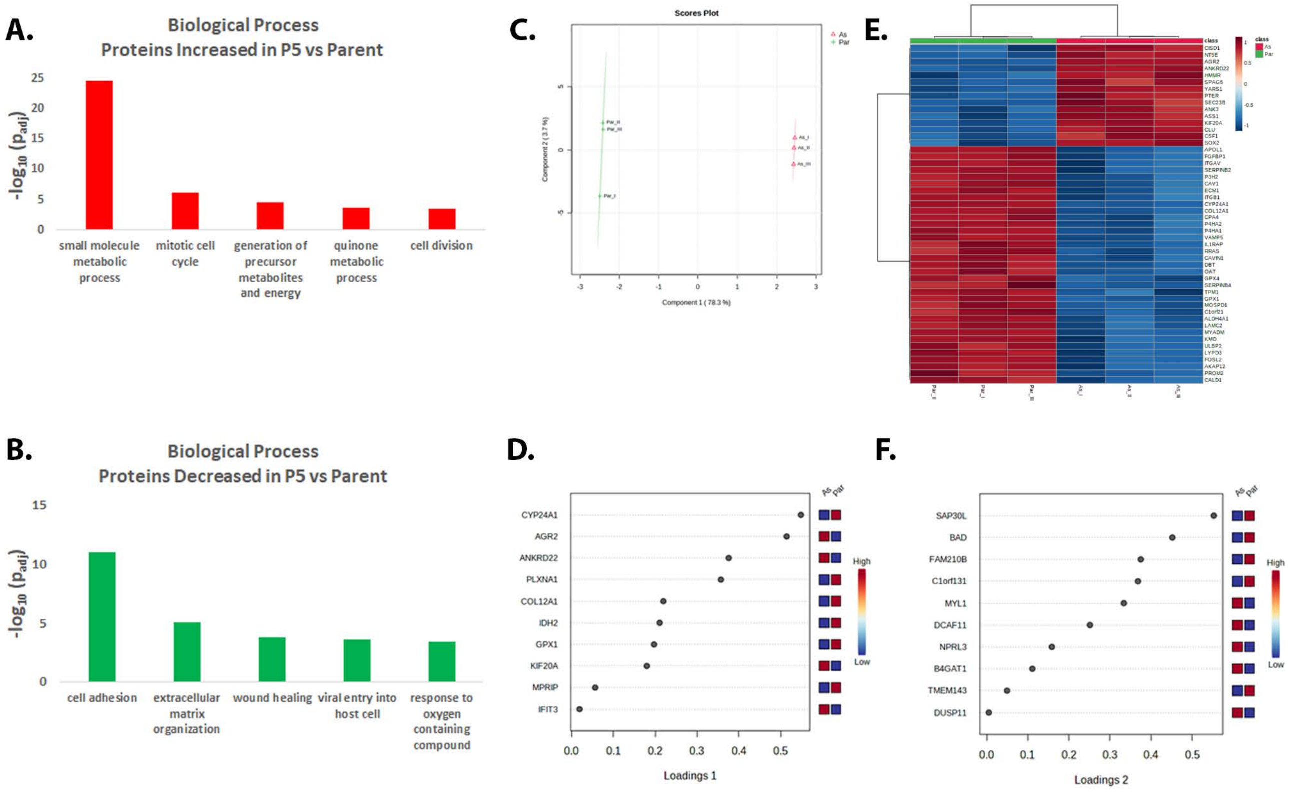

Figure S3. Proteomic analysis of UROtsa cells after 10 passages in 1 μM As^{3+}

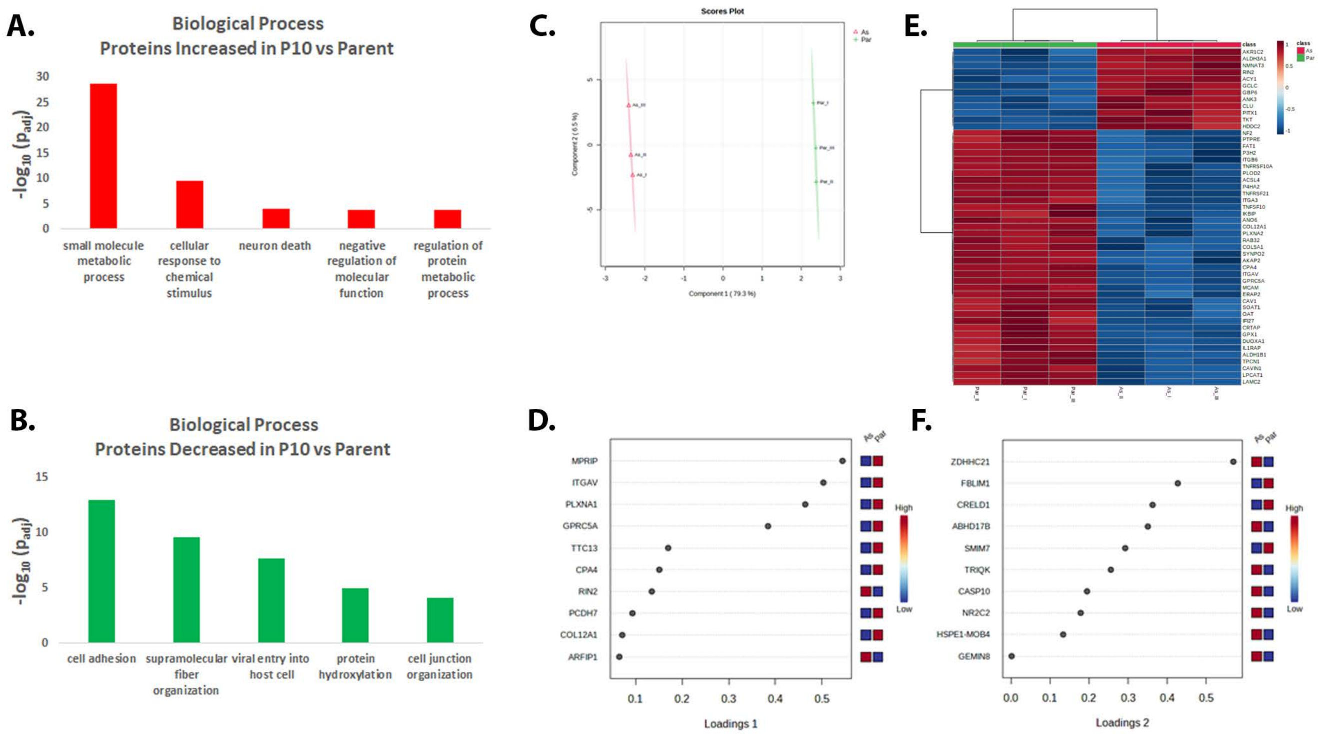

Figure S4. Proteomic analysis of UROtsa cells after 20 passages in 1 μM As^{3+}

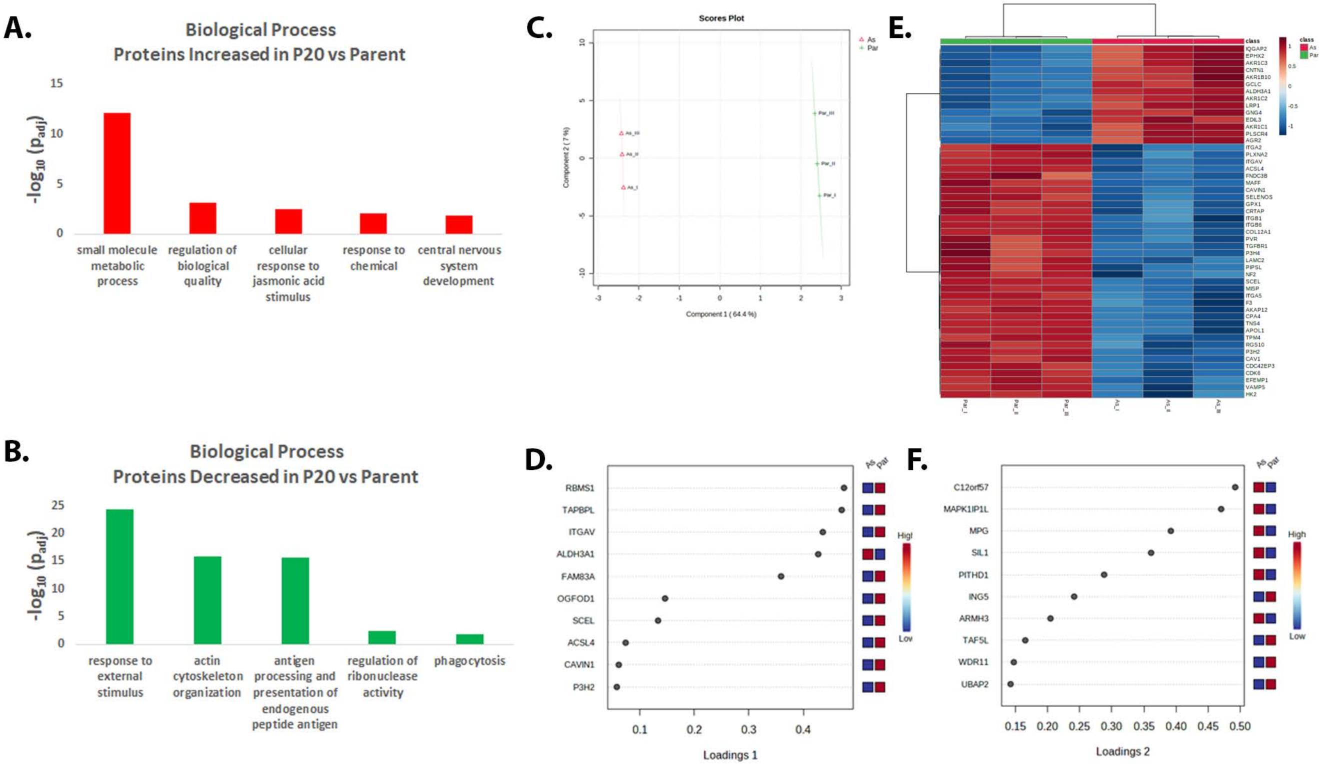

Figure S5. Morphology of UROtsa As_I cells after PVD treatments- 40X magnification

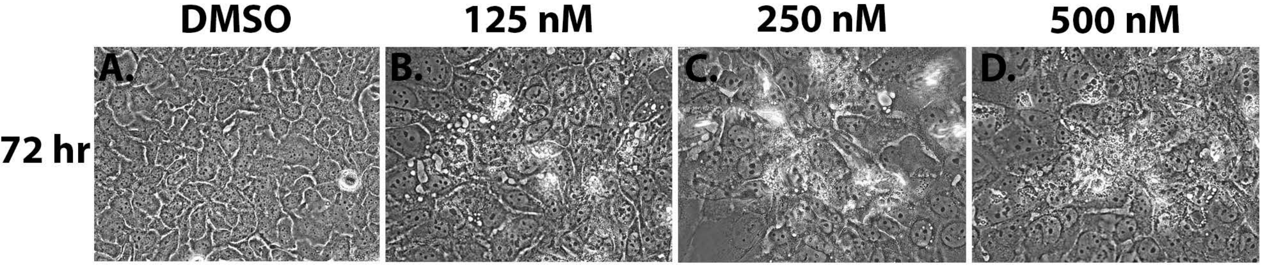

Figure S6. Morphology of UROtsa As_I cells during recovery from PVD treatments

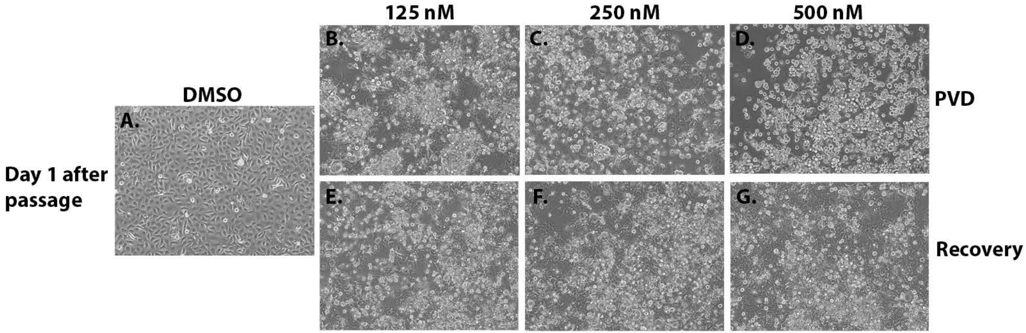

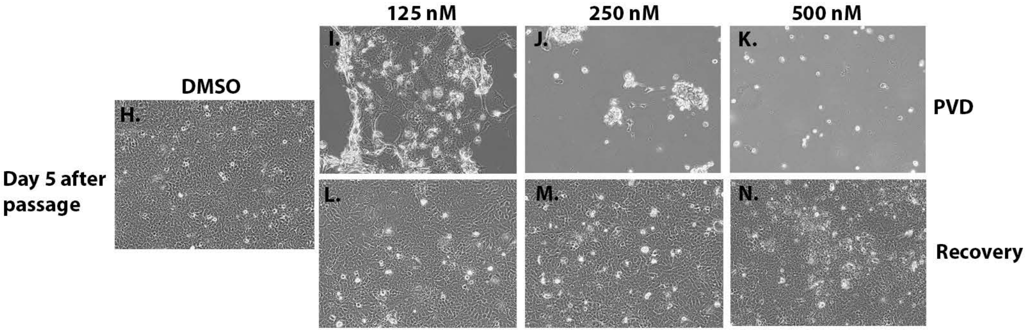

Figure S7. Morphology of UROtsa parent and As_I cells treated with PVD for 72 hours

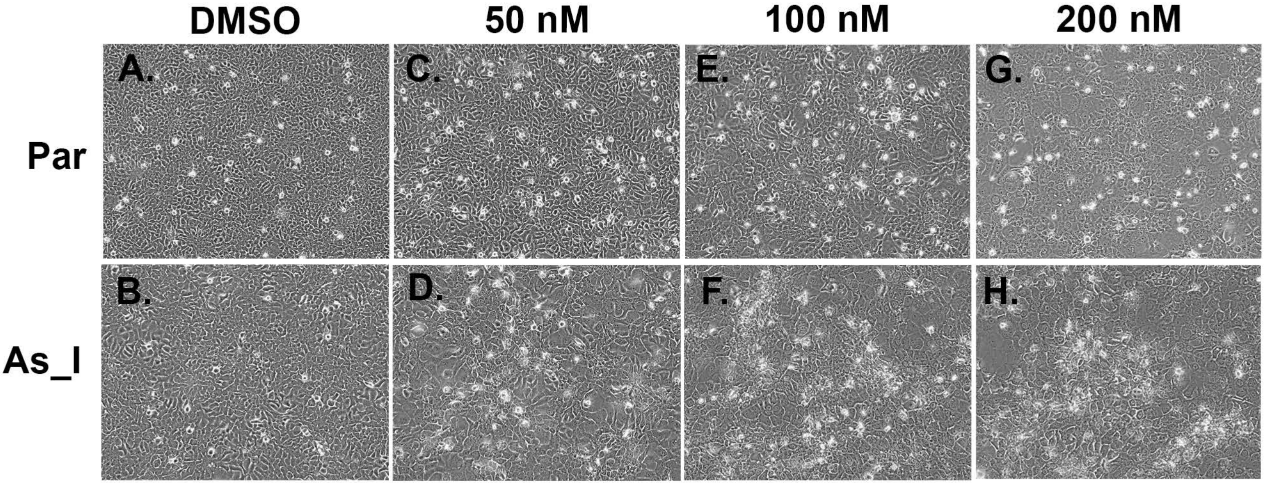

Figure S8. Viability of UROtsa parent and As_I cells treated with CIS for 72 hours

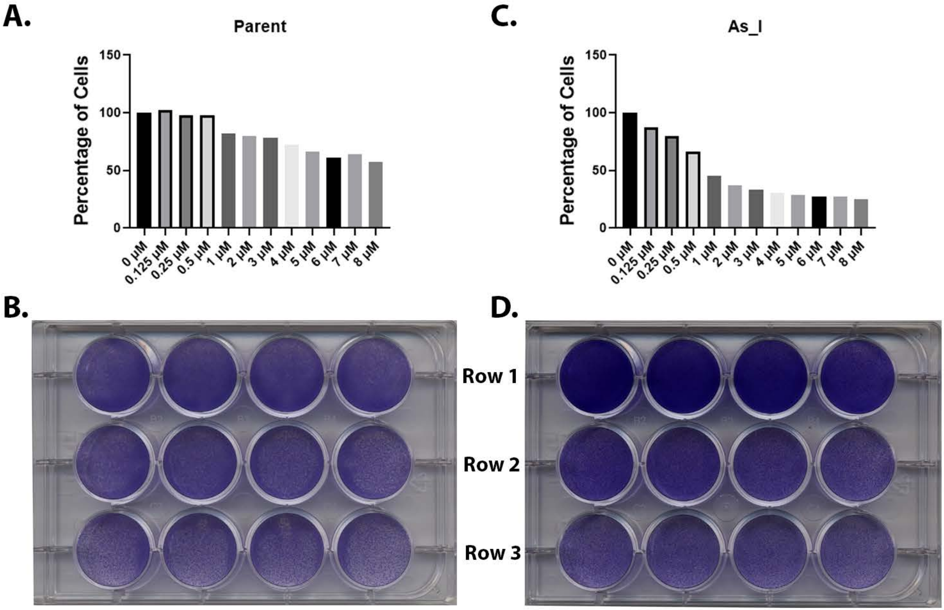

**Row 1**	**0µM, 0.125µM, 0.25µM, 0.5µM**
**Row 2**	**1µM, 2µM, 3µM, 4µM**
**Row 3**	**5µM, 6µM, 7µM, 8µM**

Figure S9. Morphology of UROtsa As_I cells after PVD and CIS treatment-20X magnfication

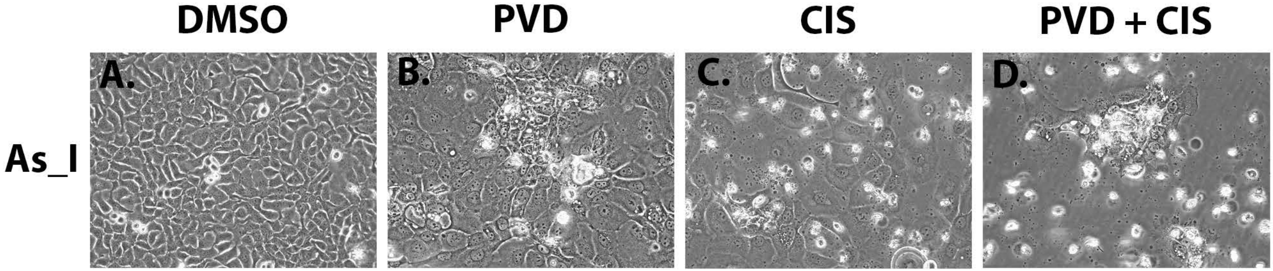

Figure S10. Additional gene expression from UROtsa parent and As_I cells treated with PVD, CIS, and PVD+CIS

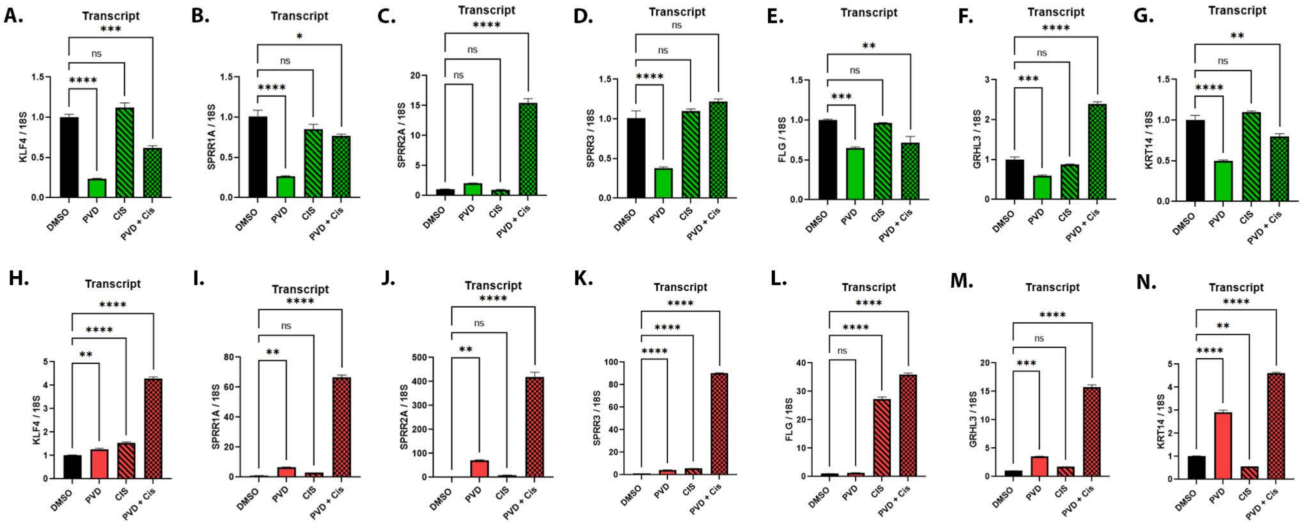

Figure S11. Additional gene expression from urospheres derived from UROtsa As_I cells

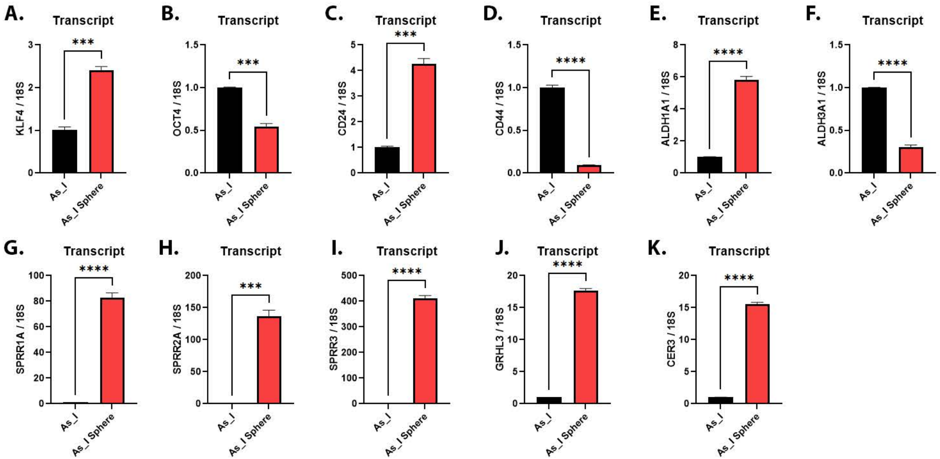

Table S1. List of primers used in the study

Genes	Product	Catalog No./unique Assay ID	Source
SOX2	PrimeTime qPCR primers	Hs.PT.58.237897.g	Integrated DNA Technologies
KLF4	PrimeTime qPCR primers	Hs.PT.58.45542593	Integrated DNA Technologies
IVL	PrimeTime qPCR primers	Hs.PT.58.39460547	Integrated DNA Technologies
KRT6A	PrimeTime qPCR primers	Hs.PT.58.26132549.g	Integrated DNA Technologies
GRHL1	PrimeTime qPCR primers	Hs.PT.58.4216223	Integrated DNA Technologies
SPRR3	PrimeTime qPCR primers	Hs.PT.58.25686932.g	Integrated DNA Technologies
DSC2	PrimeTime qPCR primers	Hs.PT.58.931954	Integrated DNA Technologies
CDH1	PrimeTime qPCR primers	Hs.PT.58.3324071	Integrated DNA Technologies
KRT16	PrimeTime qPCR primers	Hs.PT.58.40837518.g	Integrated DNA Technologies
ANXA1	PrimeTime qPCR primers	Hs.PT.56a.27417050	Integrated DNA Technologies
SPRR1A	PrimeTime qPCR primers	Hs.PT.58.39085888.g	Integrated DNA Technologies
SPRR2A	PrimeTime qPCR primers	Hs.PT.58.19341995	Integrated DNA Technologies
FLG	PrimeTime qPCR primers	Hs.PT.58.24292320.g	Integrated DNA Technologies
GRHL3	PrimeTime qPCR primers	Hs.PT.58.2379919	Integrated DNA Technologies
KRT14	PrimeTime qPCR primers	Hs.PT.58.4592110	Integrated DNA Technologies
OCT4	PrimeTime qPCR primers	Hs.PT.58.14494169.g	Integrated DNA Technologies
CD24	PrimeTime qPCR primers	Hs.PT.58.45758278.g	Integrated DNA Technologies
CD44	PrimeTime qPCR primers	Hs.PT.58.2004193	Integrated DNA Technologies
ALDH1A1	PrimeTime qPCR primers	Hs.PT.56a.38450309	Integrated DNA Technologies
ALDH3A1	PrimeTime qPCR primers	Hs.PT.56a.24823646.g	Integrated DNA Technologies
CERS3	PrimeTime qPCR primers	Hs.PT.58.14560148	Integrated DNA Technologies

*18S rRNA primers were custom ordered from IDT- Upper: CGCCGCTAGAGGTGAAATTC
Lower: TTGGCAAATGCTTTCGCTC

Table S2. Antibodies used for Western analysis

Antigen	Source	Cat. No	Protein lysate conc. (µg/µL)	Dilution
SOX2	Cell Signaling Technology	14962	1.0	1:100
IVL	Novus Biologicals	NBP2-33742	0.5	1:50
GRHL1	Novus Biologicals	NBP 1-81321	0.5	1:200
KRT16	Abcam	ab76416	0.5	1:100
ANXA1	Cell Signaling Technology	32934	0.03	1:100

Table S3. Antibodies used for Immunohistochemical analysis

Antigen	Source	Cat. No	Dilution
SOX2	Cell Signaling Technology	14962	1:100
IVL	Novus Biologicals	NBP2-33742	1:500
GRHL1	Novus Biologicals	NBP 1-81321	1:300

Supporting information legends

Figure S1. Uncropped Western blots for Figures 2, 8, and 9. (A) Displays uncropped Western blot image used in Figure 2. (B) Displays uncropped Western blot images used in Figure 8. (C) Displays uncropped Western blot images used in Figure 9.

Figure S2. Proteomic analysis of UROtsa cells after 5 passages in 1 μM As^{3+}. (A) The top five biological process pathways upregulated in UROtsa cells exposed to 1 μM As^{3+} for 5 passages. (B) The top five biological process pathways downregulated in UROtsa cells exposed to 1 μM As^{3+} for 5 passages. (C) Principle component analysis (PCA) of proteomic data comparing variability between the parent (Par_I, Par_II, Par_III) and As^{3+}-exposed (As_I, As_II, As_III) UROtsa cells at passage 5 during As^{3+}-transformation. (D) Loadings plot of proteomic data showing the main proteins responsible for driving the variability in component 1 between the parent and As^{3+}-exposed cells at passage 5. (E) Heatmap displaying the top 50 differentially expressed proteins (DEPs) between the parent and As^{3+}-exposed UROtsa cells having greater than 1.5-fold change and $p < 0.05$. (F) Loadings plot of proteomic data showing the main proteins responsible for driving the variability in component 2 between the parent and As^{3+}-exposed cells at passage 5.

Figure S3. Proteomic analysis of UROtsa cells after 10 passages in 1 μM As^{3+}. (A) The top five biological process pathways upregulated in UROtsa cells exposed to 1 μM As^{3+} for 10 passages. (B) The top five biological process pathways downregulated in UROtsa cells exposed to 1 μM As^{3+} for 10 passages. (C) Principle component analysis (PCA) of proteomic data comparing variability between the parent (Par_I, Par_II, Par_III) and As^{3+}-exposed (As_I, As_II, As_III) UROtsa cells at passage 10 during As^{3+}-transformation. (D) Loadings plot of proteomic

data showing the main proteins responsible for driving the variability in component 1 between the parent and As^{3+}-exposed cells at passage 10. (E) Heatmap displaying the top 50 differentially expressed proteins (DEPs) between the parent and As^{3+}-exposed UROtsa cells having greater than 1.5-fold change and $p < 0.05$. (F) Loadings plot of proteomic data showing the main proteins responsible for driving the variability in component 2 between the parent and As^{3+}-exposed cells at passage 10.

Figure S4. Proteomic analysis of UROtsa cells after 20 passages in 1 µM As^{3+}. (A) The top five biological process pathways upregulated in UROtsa cells exposed to 1 µM As^{3+} for 20 passages. (B) The top five biological process pathways downregulated in UROtsa cells exposed to 1 µM As^{3+} for 20 passages. (C) Principle component analysis (PCA) of proteomic data comparing variability between the parent (Par_I, Par_II, Par_III) and As^{3+}-exposed (As_I, As_II, As_III) UROtsa cells at passage 20 during As^{3+}-transformation. (D) Loadings plot of proteomic data showing the main proteins responsible for driving the variability in component 1 between the parent and As^{3+}-exposed cells at passage 20. (E) Heatmap displaying the top 50 differentially expressed proteins (DEPs) between the parent and As^{3+}-exposed UROtsa cells having greater than 1.5-fold change and $p < 0.05$. (F) Loadings plot of proteomic data showing the main proteins responsible for driving the variability in component 2 between the parent and As^{3+}-exposed cells at passage 20.

Figure S5. Morphology of UROtsa As_I cells after PVD treatments- 40X magnification. (A-D) Brightfield microscope images of As_I cells taken at 40X magnification. (A) DMSO control at 72 hours. (B) 125 nM PVD at 72 hours. (C) 250 nM PVD at 72 hours. (D) 500 nM PVD at 72 hours. Forty-eight hours prior to treatments, 100,000 cells were seeded per well of a 12-well plate and allowed to grow. The cells were then treated for 72 hours.

Figure S6. Morphology of UROtsa As_I cells during recovery from PVD treatments. (A-G) Brightfield images on first day after passaging UROtsa As_I cells that had previously been exposed to DMSO or various concentrations of PVD for 72 hours. The cells were either passaged into media containing various concentrations of PVD (PVD group) or passaged into regular media (recovery group). (H-N) Brightfield images of UROtsa As_I cells (described above) on day 5. The cells were fed fresh media only (recovery group) or fed fresh media containing various concentrations of PVD (PVD group) on day 3. All photos were taken at 10X magnification.

Figure S7. Morphology of UROtsa parent and As_I cells treated with PVD for 72 hours. Brightfield microscope images of UROtsa parent and As_I cells taken at 10X magnification. (A) parent DMSO, (B) As_I DMSO, (C) parent 50 nM PVD, (D) As_I 50 nM PVD, (E) parent 100 nM PVD, (F) As_I 100 nM PVD, (G) parent 200 nM PVD, (H) As_I 200 nM PVD. Forty-eight hours prior to treatments, 100,000 cells were seeded per well of a 12-well plate and allowed to grow. The cells were then treated for 72 hours.

Figure S8. Viability of UROtsa parent and As_I cells treated with CIS for 72 hours. Parent (A,B) or As_I (C,D) UROtsa cells were treated with various concentrations of CIS and were stained with crystal violet after 72 hours. Forty-eight hours prior to treatments, 100,000 cells were seeded per well of a 12-well plate and allowed to grow. The cells were then treated for 72 hours.

Figure S9. Morphology of UROtsa As_I cells after PVD and CIS treatment- 20X magnification. (A-D) Brightfield microscope images of As_I cells taken at 20X magnification. (A) DMSO, (B) 50 nM PVD, (C) 1μM CIS, (D) 50 nM PVD + 1 μM CIS. Forty-eight hours

prior to treatments, 300,000 cells were seeded per well of a 6-well plate and allowed to grow. The cells were then treated for 72 hours.

Figure S10. Additional gene expression from UROtsa parent and As_I cells treated with PVD, CIS, and PVD+CIS. Cells were treated for 72 hours with DMSO, 50 nM PVD, 1 µM CIS, or 50 nM PVD + 1 µM CIS treatments in parent (green bar graphs) and As_I UROtsa (red bar graphs) cells. Parent transcript levels for (A) KLF4, (B) SPRR1A, (C) SPRR2A, (D) SPRR3, (E) FLG, (F) GRHL3, (G) KRT14. As_I transcript levels for (H) KLF4, (I) SPRR1A, (J) SPRR2A, (K) SPRR3, (L) FLG, (M) GRHL3, (N) KRT14. All data is plotted as fold-change compared to the DMSO control. Gene expression was normalized to the 18S housekeeping gene. The gene measurements were performed in triplicates and the values reported are mean ± SEM. An ordinary one-way ANOVA was performed followed by a Dunnett's post-hoc test. Asterisks indicate significant differences from the DMSO control ($p < 0.05$).

Figure S11. Additional gene expression from urospheres derived from UROtsa As_I cells. As_I cells were grown as monolayer in regular T-75 flasks (As_I, black bars) or ultra-low attachment T-75 flasks (As_I spheres, red bars) in serum free media. The transcript level for (A) KLF4, (B) OCT4, (C) CD24, (D) CD44, (E) ALDH1A1, (F) ALDH3A1, (G) SPRR1A, (H) SPRR2A, (I) SPRR3, (J) GRHL3, and (K) CERS3 in As_1 cells and As_I spheres. All data is plotted as fold-change compared to the As_I cells. Gene expression was normalized to the 18S housekeeping gene. The gene measurements were performed in triplicates and the values reported are mean ± SEM. A t-test was performed to determine significance. Asterisks indicate significant differences between the As_I cells and As_I spheres ($p < 0.05$).

Table S1. List of primers used in the study

Table S2. Antibodies used for Western analysis

Table S3. Antibodies used for Immunohistochemical analysis
